# First report of gut bacterial dataset of a tribal Bhutia family from West Bengal, India

**DOI:** 10.1016/j.dib.2022.107859

**Published:** 2022-01-22

**Authors:** Souradip Basu, Kaustav Das, Mahashweta Mitra Ghosh, Rajat Banerjee, Subrata Sankar Bagchi, Sayak Ganguli

**Affiliations:** a Department of Anthropology, Bangabasi College, Kolkata 700009; b Department of Microbiology, St. Xavier's College (Autonomous), Kolkata 700016; c Department of Biotechnology, University of Calcutta, Kolkata 700019; d Dr. B.R. Ambedkar Chair, Department of Anthropology, University of Calcutta, Kolkata 700019; e Department of Biotechnology, St. Xavier's College (Autonomous), Kolkata 700016

**Keywords:** Mongolian Tribe, Bhutia, Metagenomics, GBP

## Abstract

The tribes of West Bengal are distributed in geographically distinct regions with distinctive features of their habitats and many of these tribes still practice a traditional livelihood avoiding the western diet. Hence, it is expected that their gut should remain pristine. In this study, we report the gut bacterial abundance of a Drukpa Bhutia tribal family of Lepchakha, inhabiting the hilly terrain of the Buxa region of Alipurduar district. First fecal matter was collected followed by Illumina Hiseq sequencing. Following standard protocols for metagenomic analysis, quality control (FASTQC), taxonomic profiling (QIIME, KRONA) and pathogenic load analysis were performed. This study revealed a set of core gut bacteria among which *Bacteroides* was identified to be the most abundant followed by *Bifidobacterium*, *Streptococcus* etc. Genera exhibiting lowest abundance were *Eggerthella*, *Ruminococcus*, *Enterococcus* etc. among the male, kid and female respectively. This data provides important insights into the distribution of bacterial members under study.

## Specifications Table

**Table:** 

Subject	Biological Sciences
Specific subject area	Gut Bacterial Profiling
Type of data	NGS Based Data represented in form of Pie chart and Heat Map
How the data were acquired	Illumina Hiseq Next Gen Sequencing Platform; FASTQC, QIIME
Data format	Raw FASTQ files
Description of data collection	DNA isolation and sequencing from first faecal matter of the subjects. Medical profiling of the candidates comprising of anthropometric measurements, blood pressure check-up, and dietary intake of the last 24 hours of all the participants was again performed following the procedure. Subjects were also allowed to have their regular diet.
Data source location	Institution: University of Calcutta, St. Xavier's College (Autonomous), Kolkata City/Town/Region: Kolkata Country: India
Data accessibility	https://www.ncbi.nlm.nih.gov/sra/PRJNA723462 - Bioproject Data from male: SRX10651311; Data from female: SRX10650646; Data from kid: SRX10655654 - SRA accession number

## Value of the Data

• The importance of this data stems from the fact that it is the first report of gut bacterial abundance from the Tibetan language-speaking, mountain dwellers Drukpa Bhutia tribe of Mongolian descent of Lepchakha, Buxa region of Alipurduar district of West Bengal who live in clusters separated by nearly inaccessible terrain and rely on available forest resources as well as small terrain agriculture for their livelihood.
• The studied tribe has relied upon traditional agricultural practices and is still dependent on livestock farming, locally produced vegetables and fruits for their livelihood, along with liquor and fermented dairy products, making their gut prototypical for unadulterated microbes. Moreover, these guts are still protected from overuse of medications and antibiotics, antibiotic-resistant microorganisms should be rare in the profiles [1].
• The data also demonstrates the prevalence of gut bacterial profiles shifting from father to son and mother to son, indicating parental contribution in the formation of the child's gut microbiome.

## 1 Data Description

**Fig. 1:**
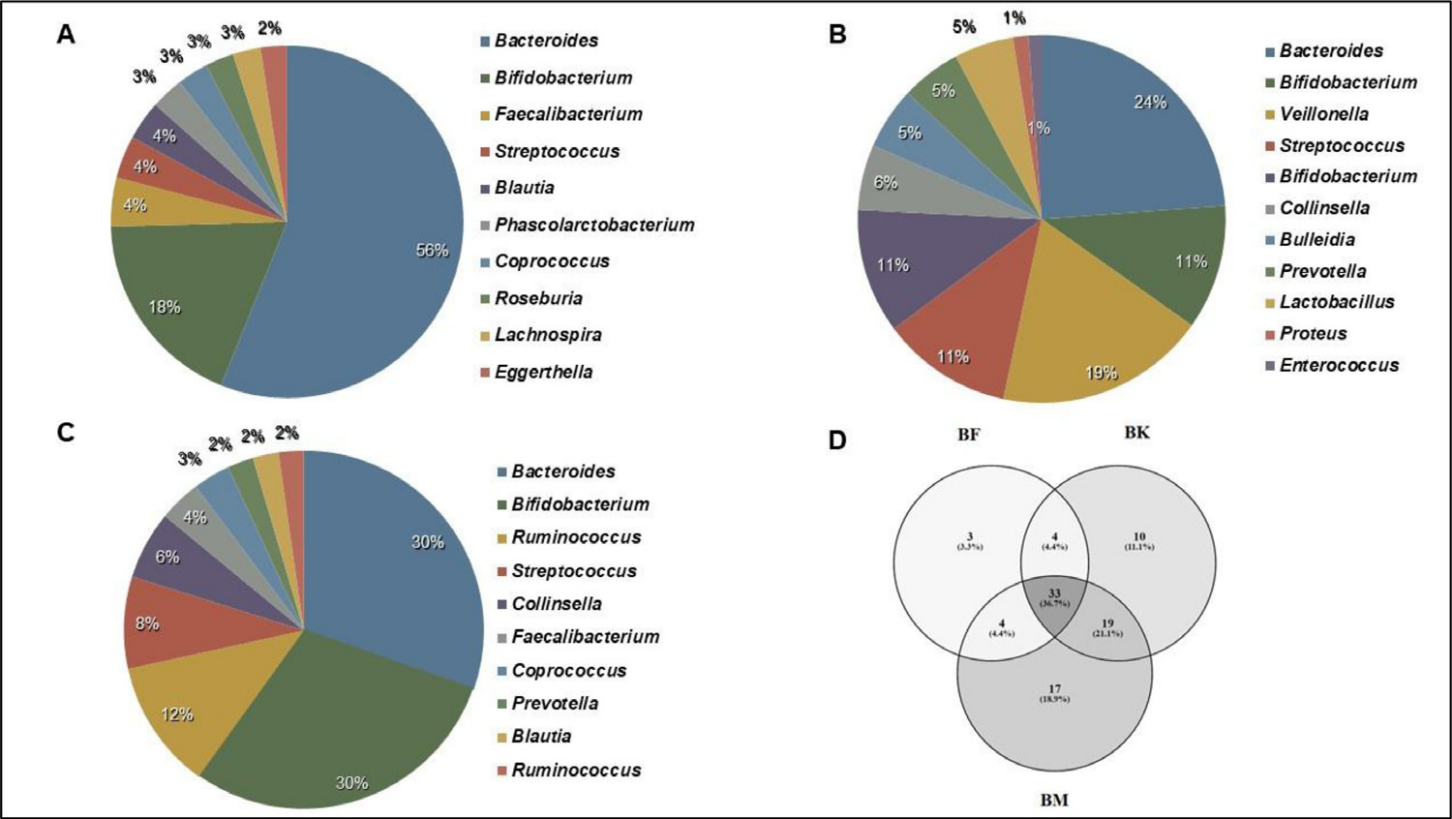
Gut Bacterial Profile of the Bhutia family under study. A Male; B: Female and C: Kid (male); D: Common taxa between the members; where BM= Male; BF= Female and BK= Child.

**Fig. 2:**
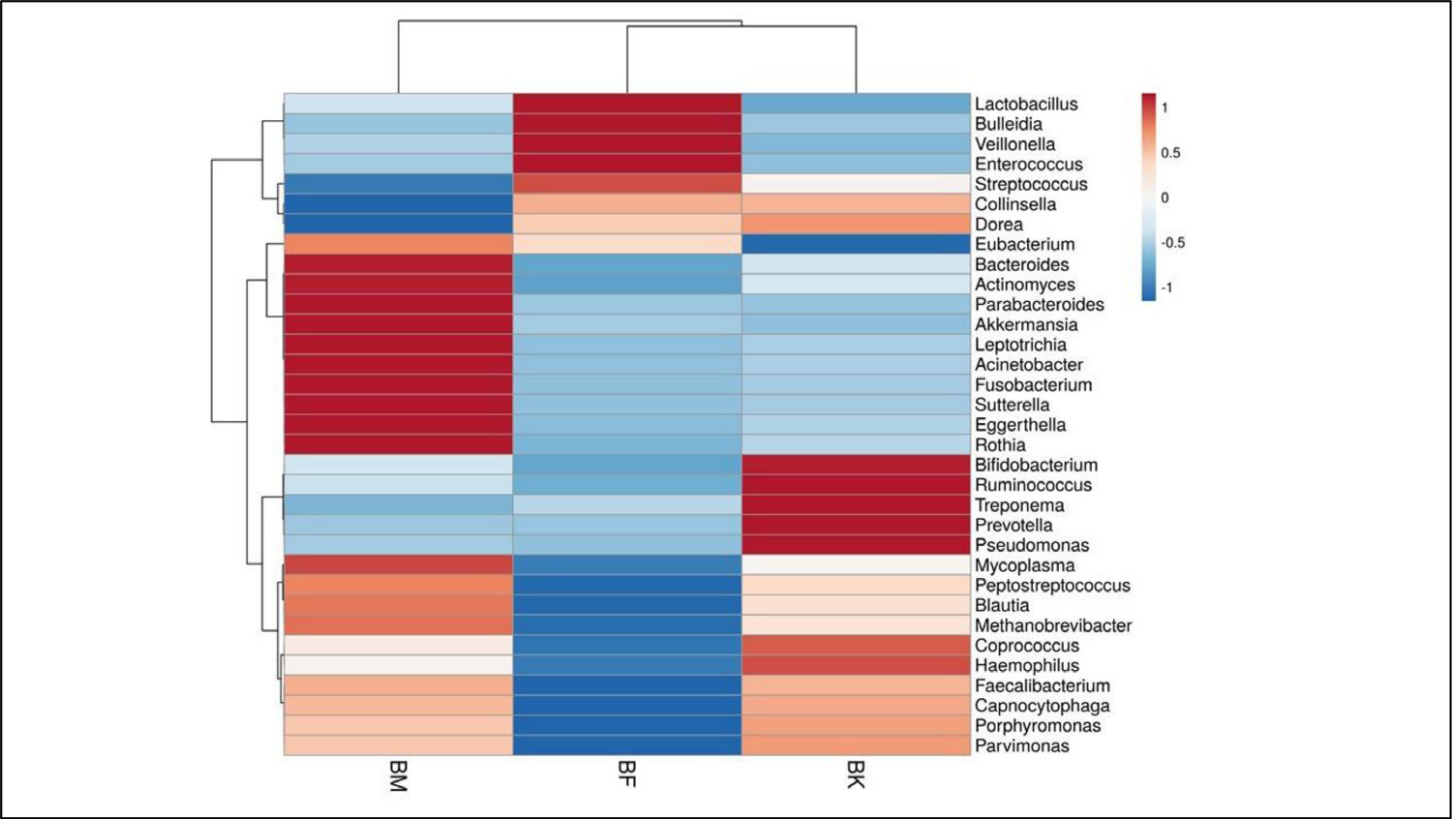
Heatmap representing the top common taxa between the members under study, where BM= Male; BF= Female and BK= Kid.

This data deals with the gut of a family belonging to the Mongolian descent tribe of West Bengal – Drukpa Bhutia. The study based on an adult Bhutia male (age: 29 years), an adult Bhutia female (age: 27 years) and their male child (Age: 5 years). Necessary permissions and informed consents were obtained from the local administration and the participants before the commencement of the study. The remoteness of the settlements of these families have been one of the major deterrents towards more such studies being conducted. The collection team hiked for three and half hours per trip and reached the settlements while passing through the buxa tiger reserve jungles. Data has been uploaded in NCBI server. The accession numbers are respectively SRX10651311, SRX10650646, SRX10655654 for Bhutia Male, Bhutia Female and Bhutia Kid. From the metagenomic analysis, we were able to obtain total 189 OTUs (Operational Taxonomic unit) belonging to the three tribal members. Alpha diversity across the three samples were 75, 45 and 69 for male, female and kid respectively. Amidst them *Bacteroides* was identified to be the common, abundant microbial member with abundances of 56%, 27% and 30% in Bhutia male, female and kid respectively. Whilst *Eggerthella, Enterococcus, Ruminococcus* etc. exhibited lowest abundance among the male, female and kid respectively (Fig. 1). Unique gut bacterial profile (GBP) of Bhutia male, female and kid exhibited *Phascolarctobacterium, Catenibacterium, Burkholderia* respectively. Following this, the common taxa between the individuals [male vs female; male vs kid and female vs kid; (Fig. 1D) were identified and the most prevalent members were analysed using a heatmap (Fig. 2).

## 2 Experimental Design, Materials and Methods

Data collection was performed through two steps:

a) Counselling and Medical Evaluation: The subjects were initially counselled on the requirement of the first faecal matter and were allowed to feed on their regular diet comprising of staple rice, puffed rice, boiled veggies with additional oil, cheese and beverages like tea for one week with sleeping proximity of average 12 hours per day. The wellness of the individuals was assured by measuring BMI (Body Mass Index) and blood pressure with the assistance of a medical professional, ensuring that the subjects were devoid of any kind of chronic diseases and morbid condition in the past six months and for 15 days.
b) Collection and sequencing: The first faecal matter was collected in sterile containers at 5.30 am in morning. The medical profiling of the candidates comprising of anthropometric measurements was performed following established procedure, and dietary intake of the last 24 hours was also noted of all the participants was again performed following the procedure. Necessary arrangements were made by packing the faecal matter in sterile containers and sealed with paraffin for transportation within 10 hours of collection to the sequencing facility. Finally, using Illumina Hiseq platform, sequencing was performed following the protocol described in [2] with the Bioinformatics pipeline as previously reported [3]. Following this, utilization of standard bioinformatics pipeline [4], Krona, SILVA [5] and Greengenes [6] databases were used to first quality check the data followed by identification of the most abundant taxa.

## Ethics Statement

This research has been carried out in accordance with The Code of Ethics of the World Medical Association (Declaration of Helsinki) and was approved by the Research and Ethics Committee of Bangabasi College, University of Calcutta (No. 002/2017).

## CRediT Author Statement

**Sayak Ganguli, Souradip Basu, Subrata Sankar Bagchi:** Conceptualization, Methodology, Software; **Kaustav Das, Souradip Basu, Mahashweta Mitra Ghosh:** Data curation, Writing- Original draft preparation; **Souradip Basu, Sayak Ganguli:** Visualization, Investigation; **Mahashweta Mitra Ghosh, Rajat Banerjee, Subrata Sankar Bagchi:** Supervision; **Kaustav Das, Souradip Basu:** Software, Validation; **Souradip Basu, Sayak Ganguli:** Writing- Reviewing and Editing.

## Declaration of Competing Interest

The authors declare that they have no known competing financial interests or personal relationships that could have appeared to influence the work reported in this paper.
